# Water Softening for Institutions

**Published:** 1912-06-22

**Authors:** F. P. Carroll

**Affiliations:** Assistant Secretary, Charing Cross Hospital.


### WATER SOFTENING FOR INSTITUTIONS.

### The Type and Cost of Various Appliances Compared.

By F. P. CARROLL, Assistant Secretary, Charing Cross Hospital.

The various ill-effects of hard water have already been touched upon in The Hospital (April 27, p. 78, and May 18, p. 188), where it was pointed out that in laundries a pound of soap or more is absolutely wasted for each degree of hardness in the water, and that a quarter of an inch of scale inside a boiler entails a consumption of 10 tons of coal extra per week. It may be added that hard water is equally objectionable in cooking, as the lime salts deposit upon the food and harden the tissues. Ten ounces of tea made with soft water are said to be as strong as 16 ounces made with hard water, while soup made with the latter requires an additional amount of meat, and the cooking takes a longer time. In baking the dough rises better and is of a lighter colour where soft water is used, while a hard water makes vegetables become very dark.

It is apparent, therefore, without pressing the point further, how extremely desirable it is that water should be softened for either or all of its three chief purposes—boiler-feed, laundry work, and domestic uses. The economy thus to be effected is of the first importance, while the remarkably pleasant feeling arising from the use of soft water in baths and hand-basins remains as an additional advantage.

The first attempts to soften water on a scientific basis appear to have been made about seventy years ago by Professor Clark, after whom the well-known "Clark's process" is named. The Professor patented his process in 1840, the basis of it being the addition of lime water. This, however, removed only the *temporary* hardness, and it is, of course, the *permanent* hardness which is of the more importance, as being the more harmful. This had to be removed independently by the addition of soda. Since Clark's time his plan has undergone many modifications and improvements, while remaining in essence the same; but until quite recently the cost of plants for adding lime and soda, simple though the principle appears to be, has been almost prohibitive. The stumbling-block has been the difficulty of diffusing the softening agent in its right proportions throughout the mass of the water, while the necessity for very large tanks in which the precipitation might take place has added very much to the cost of the outfits. There are, indeed, some types of water softener which claim to obviate the necessity for chemicals, the method used being by boiling the water; but these are just as expensive as the others, and they remove only the temporary hardness, being obliged to have resource to chemicals to remove the sulphates.

So far as boiler-feed water is concerned, there are, of course, many methods of dealing with the precipitates after the hard water has been run into the boiler, and of thus circumventing the necessity for a softener. On the familiar analogy of a marble in the domestic kettle these methods include the placing in the boiler of wires, chains, brushes, twigs, fibres, peat, bran, potato-pulp, sea-weed, starch, chestnuts, bacon, and treacle; while there are a large number of proprietary compounds with which to paint the inside of the boiler, these having the effect, not of preventing the incrustation, but of softening it, and thus making it easier to remove. These methods have a certain amount of success, but the labour and expense attaching to them are somewhat considerable, and the accumulation of foreign matter in the boiler is very undesirable. Moreover, such methods do not touch the question of water supply for laundries and domestic purposes. An adequate softening plant is, therefore, the only scientific solution of the problem, and so long as the initial outlay is not too high, it is the only one possible from the double standpoint of economy and efficiency.

The table on page 307 gives a comparison between six types of water softener. In each case the maker has based his estimate on a flow of 1,000 gallons per hour and on a water of 15 degrees hardness, while the price given is apart from the cost of fitting up (which, of course, would include any structural alterations that might be necessary to accommodate the plant, according to circumstances). The expense of upkeep is reckoned on a basis of 12 hours' working per day.

It will be seen from the table that the addition of lime and soda forms the basis of nearly all the plants, this being true probably of those in which the constituents of the agent are not given. Several of the systems are well known, and are of many years' standing. Number 3, in which an estimated price could not be given, requires very large settling tanks, and this is a grave consideration in the case of charitable institutions. Number 2 is one of the most recent, and is distinctive in so far that no liquid solution is added, but the softening material requires the addition of chemicals from time to time to stimulate its action. Number 6 stands out as the cheapest as well as the most recent, but it has not undergone as long a test of time as most of the others. Its cheapness is due to the distinctive method it employs of mixing the agent with the water, which obviates the necessity for any large tank. The solution used is understood to be based upon caustic soda prepared by a secret process which renders it both cheaper and more efficient. The plant is of quite small size, and it claims to reduce the water to any desired degree of hardness.

| No. | Brief description | Cost | Cost of upkeep per ann. |
|---|---|---|---|
| 1 | Lime and soda added by tipping bucket; precipitates filtered ... | £150 | £10 |
| 2 | Water runs through mass of material; precipitates washed out periodically ... ... ... | £150 | £23 |
| 3 | Lime and soda added in large tank, precipitates allowed to settle, and water decanted ... | — | — |
| 4 | Lime and soda added by tipping bucket; precipitates filtered ... | £150 | £6 |
| 5 | Secret solution added by water-wheel; precipitates filtered ... | £120 | £15 |
| 6 | Secret solution, steam, and water mixed simultaneously in patent valve; precipitates filtered ... | £30 | £4 |

The cost of softening plants is, generally speaking, cumulative, an expensive plant being a big plant—a big plant requiring structural alterations, and structural alterations adding greatly to the initial outlay. (A case is within the writer's knowledge in which the maker's estimate for a plant was £240, while the cost of fitting it up was reckoned by the institution's engineer to amount to at least as much again.) Conversely, a cheap plant is a small plant, and a small plant can usually be fitted into existing accommodation. Similarly, the cost of upkeep is as a rule proportionate to the initial outlay.

On the question as to what degree of hardness is desirable in a water opinions differ. The writer was recently visiting a large institution near London in which 60,000 gallons of water a day are consumed. Two wells have been sunk and a big softening plant installed to treat the whole supply, but the water is not reduced below 6 degrees (and this at a cost of about £180 per annum). This the engineer considered to be sufficient, asserting that if water were 0 degrees hard for laundry work it would be impossible to get the soap out of the clothes. However this may be, it is probable that most engineers would not leave their water supply at 6 degrees of hardness if they were able to bring it lower, the general opinion being that the lower the hardness of water for laundry purposes the better, while water for boiler-feed should retain some 2 or 3 degrees as a safeguard against the corrosion of the internal fittings.

